# Bacterial and fungal pattern recognition receptors in homologous innate signaling pathways of insects and mammals

**DOI:** 10.3389/fmicb.2015.00019

**Published:** 2015-01-28

**Authors:** Bethany A. Stokes, Shruti Yadav, Upasana Shokal, L. C. Smith, Ioannis Eleftherianos

**Affiliations:** Insect Infection and Immunity Laboratory, Department of Biological Sciences, The George Washington UniversityWashington, DC, USA

**Keywords:** recognition receptors, innate immunity, immune signaling, insects, mammals, pathogens

## Abstract

In response to bacterial and fungal infections in insects and mammals, distinct families of innate immune pattern recognition receptors (PRRs) initiate highly complex intracellular signaling cascades. Those cascades induce a variety of immune functions that restrain the spread of microbes in the host. Insect and mammalian innate immune receptors include molecules that recognize conserved microbial molecular patterns. Innate immune recognition leads to the recruitment of adaptor molecules forming multi-protein complexes that include kinases, transcription factors, and other regulatory molecules. Innate immune signaling cascades induce the expression of genes encoding antimicrobial peptides and other key factors that mount and regulate the immune response against microbial challenge. In this review, we summarize our current understanding of the bacterial and fungal PRRs for homologous innate signaling pathways of insects and mammals in an effort to provide a framework for future studies.

## INTRODUCTION

Innate immune systems serve as the first line of defense to protect host organisms from a wide variety of infections through mechanisms that can be activated rapidly upon recognition of a foreign threat (Kumar et al., 2011). With the addition of an adaptive immune system, mammals are able to confer specific immunological memory through the production of antigen-specific antibodies secreted from memory B cells that can strengthen the immune response to a secondary infection (Palm and Medzhitov, 2009). However, for the 5–10 million species of metazoans lacking an adaptive immune system, the diverse innate signaling pathways are sufficient and mandatory to eradicate infections. Insects have proven capable of inducing clearance of microbial burdens that can be lethal to most mammals (Silverman and Maniatis, 2001; Ligoxygakis, 2013). Even in the absence of an adaptive immune system homologous to those of mammals, insects can nonetheless discriminate and recognize particular infections and induce the release of molecules that are efficient for controlling specific classes of intruders (Ferrandon et al., 2007).

The Toll, Immune deficiency (Imd), and Janus Kinase and Signal Transducer and Activator of Transcription (JAK/STAT) pathways are the three major signaling pathways responsible for the distinctly robust innate immune response of insects (Lemaitre and Hoffmann, 2007). Each pathway individually recognizes a pathogen, or binds a recognition signal, and induces the transcription of a specific set of immune-related genes that encode peptides, which can either target the pathogen for degradation or function as signaling molecules to amplify the immune response. The Toll pathway is mainly accountable for the detection of and response to Gram-positive bacteria and fungal infections, whereas the Imd pathway is required for responses to Gram-negative bacterial infections (Lindsay and Wasserman, 2014; Myllymäki et al., 2014). The JAK/STAT pathway is activated by infection or septic injury, which are detected by a variety of immunological effectors in insects and by the release of cytokines in mammals (Agaisse and Perrimon, 2004). Together, Toll and Imd pathways constitute the humoral immune response that functions through the production of antimicrobial peptides (AMPs). These are small cationic molecules are produced in the fat body (equivalent to mammalian liver) and hemocytes (equivalent to mammalian white blood cells) and subsequently released into the hemolymph (Bulet and Stöcklin, 2005). AMPs degrade and induce lysis of bacteria and fungi by interaction with intracellular and cell wall components. The genes encoding the AMPs Drosomycin and Diptericin are the major outputs of Toll and Imd signaling, respectively. These two genes are transcribed in tandem with several other AMP-coding genes in response to microbial infection (Imler and Bulet, 2005). In addition, JAK/STAT signaling induces the production of molecules critical to the inflammatory immune response of insects, the result of which are largely in the form of cytokines, antimicrobial molecules, and proteins involved in innate cellular responses, such as melanization and phagocytosis (Myllymäki and Rämet, 2014).

Mammals induce a rapid innate immune response that converts the recognition of a pathogen into the recruitment of leukocytes and inflammatory cells to the site of infection in order to eradicate the pathogen and simultaneously to trigger the production of antibodies (Iwasaki and Medzhitov, 2010). There are striking similarities between insect and mammalian innate immune signaling pathways. Mammals use Toll-like receptors (TLRs), named for their homologous leucine-rich repeat structures that are also present in insect Toll, to activate antibacterial and fungal immune responses (Kumar et al., 2009; Chtarbanova and Imler, 2011; Szatmary, 2012). In addition, the insect Imd pathway is homologous to the mammalian Tumor Necrosis Factor Pathway (TNF), and JAK/STAT pathways in insects and mammals are homologs (O’Shea and Plenge, 2012; Myllymäki and Rämet, 2014; Myllymäki et al., 2014). In comparing the signal transduction throughout each homologous pathway, a pattern becomes quite evident; intracellularly, insects and mammals possess similar signaling components, but the terminal molecules the initiating receptors and the molecules that are produced as a result of gene expression vary considerably (Imler, 2014). While mammals do activate innate signaling to produce and secrete acute phase antigen-attacking cells, such as macrophages and neutrophils, the goal of the mammalian innate immune response is to form an initial inflammatory reaction to the invader and to communicate to the lymphocytes to initiate the adaptive immune system priming and activation. The innate immune system of insects leads to the production of AMPs that fight the infection, whereas mammals generate cytokines and chemokines that amplify the immune response and recruit antigen-presenting cells that induce antibody production (Ganesan et al., 2011; Schenten and Medzhitov, 2011).

This review will focus on the innate pattern recognition receptors (PRRs) responsible for detecting bacterial and fungal infections. In Toll, Imd, JAK/STAT, and the mammalian counterparts, the PRRs are imperative for inducing the stimulatory signal to the intracellular mechanisms. Between mammals and insects, the structure and function of many of the receptors are very different, even within homologous pathways. In certain pathways, such as Toll, the presence of extracellular circulating receptors for pathogens is critical to mount the necessary response, while mammalian counterparts do not possess homologous mechanisms (Lindsay and Wasserman, 2014). In the Imd pathway, the receptors are not homologous but are linked into identical intracellular pathways that promote similar processes for the organism (Kleino and Silverman, 2014). The JAK/STAT pathway shows the most homology with regard to the receptors involved, indicating a high level of conservation for this pathway across phyla (Myllymäki and Rämet, 2014). Here we will integrate the current information on the major receptor proteins in insect and mammalian innate homologous pathways and describe their functional differences and similarities. Detailed characterization of the PRRs will elucidate the evolutionary conservation of innate signaling and function among invertebrate and vertebrate organisms.

## TOLL AND TOLL-LIKE PATHWAY RECOGNITION RECEPTORS FOR BACTERIAL DETECTION

**FIGURE 1:**
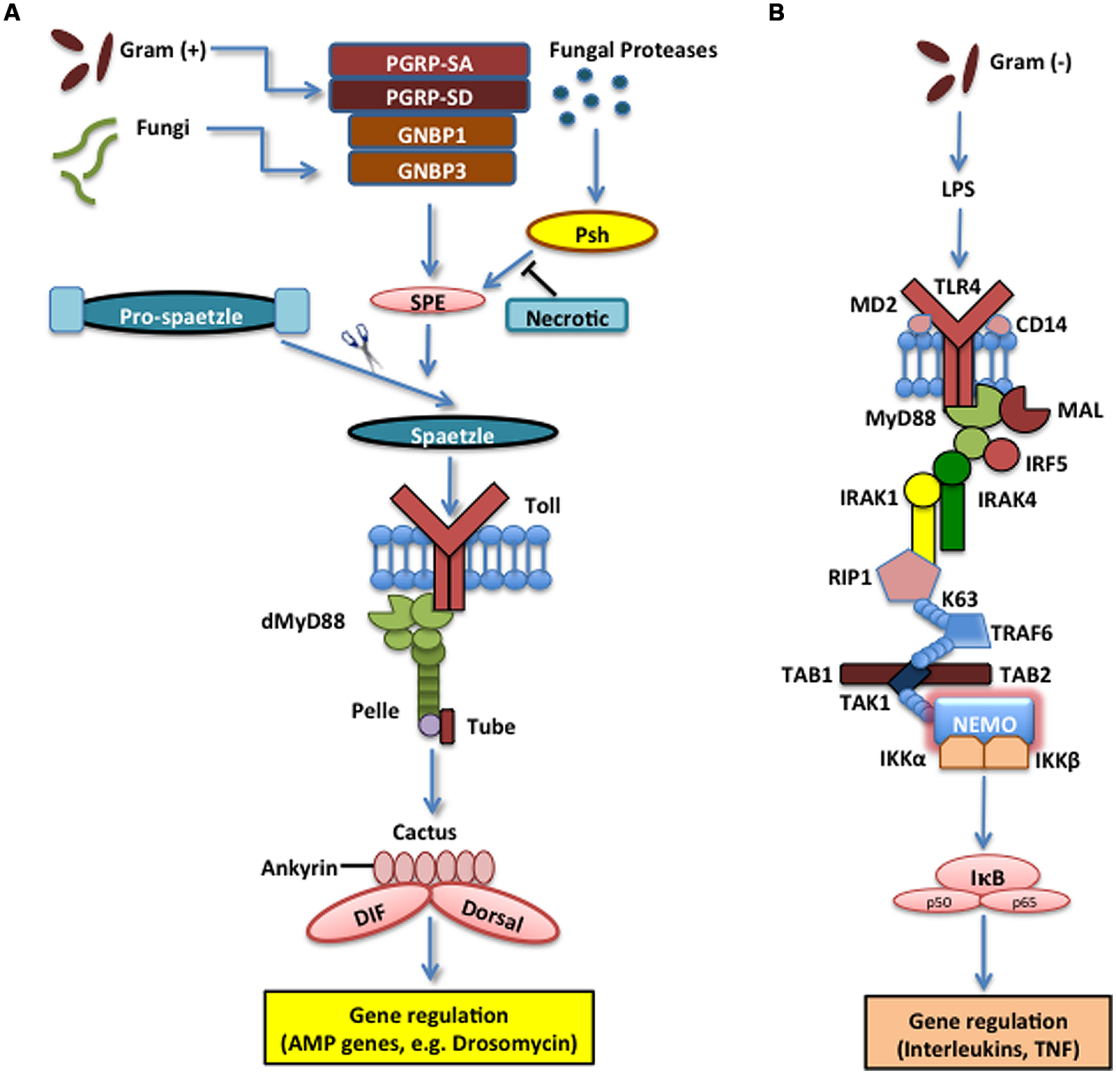
**The Toll pathway in the fruit fly and the Toll-like receptor (TLR) 4 pathway in the mouse. (A)** The Toll pathway in *Drosophila melanogaster* mainly detects fungi and Gram-positive bacteria. The Toll receptor is triggered upon binding by the cleaved form of the cytokine Spaetzle, which is processed by Spaetzle-processing enzyme (SPE) and other serine proteases that are regulated by the pathogen recognition peptidoglycan recognition proteins (PGRP) PGRP-SA, PGRP-SD, GNBP1, and GNBP3. Serine protease Persephone (Psh) is activated by virulence factors secreted by entomopathogenic fungi and is regulated by Necrotic, a Psh inhibitor. Toll receptor activation results in the recruitment of adaptor proteins in the cytoplasm including myeloid differentiation primary response 88 (dMyD88), Tube, and Pelle, which promotes signaling to Cactus and its ankyrin-repeat domains. Cactus is normally bound to the Nuclear Factor kappa B (NF-κB) transcription factors Dorsal-related Immunity Factor (DIF) and Dorsal, but upon activation of the signaling pathway, it is phosophorylated, dissociated from DIF or Dorsal and degraded. These signaling events result in the nuclear translocation of DIF or Dorsal that induce the transcriptional upregulation of antimicrobial peptide (AMP) genes, such as Drosomycin. **(B)** TLR4 receptor in *Mus musculus* functions together with Lymphocyte Antigen 96 (MD2) and Cluster of Differentiation 14 (CD14) to detect lipopolysaccharides (LPS) from Gram-negative bacteria. MyD88 is recruited with Interleukin-1 receptor-associated kinases 1 and 4 (IRAK1, IRAK4), receptor-interacting protein 1 (RIP1) and Tumor Necrosis Factor (TNF) receptor associated factor 6 (TRAF6). The latter ubiquitinates itself to recruit Transforming Growth Factor beta (TGF-β) activated kinase 1 (TAK1) and TAK1-associated binding proteins 1 and 2 (TAB1 and TAB2), which result in the activation of the IκB kinase (IKK) complex that phosphorylates the Inhibitor of NF-κB (IκB). This leads to the release of NF-κB that translocates to the nucleus and initiates the transcriptional induction of inflammatory and immune response related genes.

The Toll pathway in insects is mainly responsible for the recognition of fungi and Gram-positive bacteria and the induction of certain AMPs that are secreted into the insect hemolymph (Lemaitre and Hoffmann, 2007; Tsakas and Marmaras, 2010). Toll is a transmembrane protein composed of extracellular leucine-rich repeat modules and a cytoplasmic Toll-Interleukin-1-receptor (TIR) domain that initiates signaling. It was originally characterized based on its function in the dorsal-ventral pattern formation in the *Drosophila* embryo, but it was subsequently shown that Toll is also required for innate immune signaling (Hashimoto et al., 1988; Lemaitre et al., 1996). *Drosophila* Toll does not interact directly with microbial structures, but instead receives signals from recognition proteins in the hemolymph that converge a signal of microbial presence on Spaetzle, which is the Toll ligand (Weber et al., 2003). Upon Gram-positive bacterial infection, Toll relies on the function of three pathogen recognition proteins that detect the bacteria and trigger a serine protease cascade that activates the Spaetzle-processing enzyme (SPE) that cleaves Spaetzle into the fragment that binds Toll. This induces Toll to initiate intracellular signaling that recruits adaptor proteins, myeloid differentiation primary response 88 (MyD88), Tube, and Pelle [an interleukin-1 receptor-associated kinases (IRAK) ortholog], and signals through a poorly defined pathway to Cactus [inhibitor of kappa B (IκB) homolog], which is bound to the Rel homology domains of the transcription factors Dorsal-related Immunity Factor (DIF), Nuclear Factor κB (NF-κB) homolog, and Dorsal through its six ankyrin-repeats. Cactus phosphorylation and subsequent ubiquitination and degradation by the proteasome leads to the release of DIF that moves to the nucleus and induces the transcriptional activation of the AMP genes (Valanne et al., 2011; Lindsay and Wasserman, 2014). Without the initial signaling from the extracellular protease cascade and activation of Toll, the intracellular reactions cannot induce production of antibacterial proteins, rendering the insect susceptible to infection (**Figure 1A**).

The first PRR identified that acts prior to Toll activation, and functions to detect Gram-positive bacteria in the hemolymph of the fly was the peptidoglycan recognition protein SA (PGRP-SA; Werner et al., 2000; Michel et al., 2001). At the time, the relationship between Spaetzle and Toll was known, as well as most of the intracellular signaling components leading to AMP synthesis, but the extracellular receptors had not been identified. Screening 2,500 independent *Drosophila* cell lines, each carrying a mutation, led to the identification of the gene, *semmelweis (seml)*, because the mutant line was deficient in the production of drosomycin, but could produce diptericin in response to Gram-negative bacteria (Michel et al., 2001). The lack of production of an AMP regulated by the Toll pathway, but not an AMP regulated by the Imd pathway suggested important implications of Toll function. Sequence analysis of *seml* demonstrated that the gene encodes PGRP-SA, which is characterized as a receptor protein that binds to peptidoglycan (PG) and is found in the hemolymph, and it functions to detect Gram-positive bacterial cell wall components prior to Toll activation. Fly mutants for PGRP-SA/*seml* are highly susceptible to Gram-positive bacteria, but their susceptibility to Gram-negative bacteria is unaffected and similar to wild-type controls, further confirming the importance of PGRP-SA as a recognition molecule that functions in the Toll pathway (Michel et al., 2001). Further study of the structure of PGRP-SA identified an extended surface groove within the protein that is lined with residues that make it bind specifically to PGRP-SA (Chang et al., 2004). Mutational analysis revealed that this surface of the protein functions as a PG-binding groove and one residue in particular, Ser158, acts as a critical region for interaction with Lysine (Lys)-type PG, a component specific to Gram-positive bacterial cell walls. PGRP-SA has also been identified as an intrinsic L,D-carboxypeptidase that functions during infections with Gram-negative bacteria, in which the protein binds diaminopimelic acid (DAP)-type PG and induces hydrolysis (Chang et al., 2004). However, PGRP-SA does not have any activity toward bacteria with the Lys-type PG, indicating potential activation of both the Toll and the Imd pathways (Chang et al., 2004). Therefore, PGRP-SA is able to bind to Lys-type bacteria due to the ionic interactions with the serine residue, while also having a minimal function in fragmenting DAP-type PG for Imd recognition.

There are two additional insect protein recognition receptors, Gram-negative binding protein 1 (GNBP1) and PGRP-SD, that function in cooperation with PGRP-SA. GNBPs were first isolated from the silkworm, *Bombyx mori*, but homologous GNBPs have been found in *Drosophila* and vertebrates (Lee et al., 1996). In particular, GNBP1 can bind specifically to lipopolysaccharides (LPS) and β-glucan structures in either a soluble or membrane-bound glycosylphosphatidylinositol (GPI)-anchored form (Kim et al., 2000). In addition to binding to these pathogen associated molecular patterns (PAMPs), GNBP1 has hydrolytic activity toward Lys-type PG, which it hydrolyses into smaller fragments upon infection with certain Gram-positive bacteria, such as *Enterococcus faecalis* (Wang et al., 2006). However, it does not hydrolyze PGs from other Gram-positive bacteria, such as *Staphylococcus aureus* or *Bacillus subtilis* (Wang et al., 2006). The rate-limiting factor is the level of interaction between these PG fragments and PGRP-SA as demonstrated by *GNBP1* mutants that can induce drosomycin production compared to *PGRP-SA* mutants that cannot. The change in drosomycin production between *GNBP1* and *PGRP-SA* mutants indicates that GNBP1 can hydrolyze Lys-type PG and releases the fragmented monomers into the hemolymph. However, the absence of PGRP-SA to bind PG and relay the signal to Toll increases fly sensitivity to infection (Wang et al., 2006). In addition, GNBP1 and PGRP-SA form a complex that is enhanced by the presence of bacterial infection, further suggesting that for effective induction of Toll, dual activation of PGRP-SA and GNBP1 is critical. This activation is based on the innate ability of these proteins to recognize a minimal structure of the disaccharide of N-acetyl glucosaminyl (GlcNAc) linked to *N*-acetylmuramic acid (MurNAc) with peptide chain crosslinkers, which contain the Lys-type residues in the third position of the stem peptide (Filipe et al., 2005). In summary, Lys-type residues are specifically recognized by GNBP1 circulating in the hemolymph and upon activation, GNBP1 binds and hydrolyzes the PG cell wall of the bacteria to produce fragments. PGRP-SA binds to the PG fragments and relays the signal to that serine protease cascade that cleaves Spaetzle to generate the cytokine-like ligand of Toll, which initiates the cellular signaling to induce the production of AMPs in response to bacterial infection.

Although the PGRP-SA/GNBP1 complex is a very effective detection system leading to the activation of Toll, certain Gram-positive bacteria activate the Toll pathway in a PGRP-SA/GNBP1-independent manner, indicating an alternative bacterial recognition mechanism. Loss-of-function mutants for another short extracellular PGRP, PGRP-SD, exhibit extreme susceptibility to Gram-positive bacteria that is exacerbated in PGRP-SA/GNBP1 mutant phenotypes (Bischoff et al., 2004). This suggests a cooperative relationship with the previously identified complex to enhance the detection of Gram-positive bacteria, but not fungal pathogens. From studies on PGRP-SD, the mechanism of action is not fully known but PGRP-SD may cluster with the PGRP-SA/GNBP1 complexes to induce effective binding to bacterial cell walls or it might act independently under specific circumstances (Bischoff et al., 2004). Together, PGRP-SA, GNBP1, and PGRP-SD mount an effective recognition process toward Gram-positive bacteria that allows for Toll activation and signaling and the induction of AMPs with antibacterial/antifungal properties. Interestingly, PGRP molecules in mammals do not participate in the detection of Gram-positive bacteria. It has been hypothesized that mammalian PGRPs are more functionally similar to AMP due to their ability to target and hydrolyze specific Gram-positive bacteria and inhibit their growth through direct interaction (Liu et al., 2000).

Mammals express TLRs that are highly homologous to insect Toll receptors based on the conserved leucine-rich repeats, the TIR domains, as well as the propensity to bind to specific pathogens (Rock et al., 1998). The critical difference between Toll and TLR is the level of interaction with the pathogen. In *Drosophila*, Toll receptors only bind cleaved Spaetzle, which is only produced as the result of pathogen recognition by the receptors described above. Mammalian TLRs interact with the pathogen directly in conjunction with other surface proteins and co-receptors (Wright et al., 1990; Park et al., 2004). On the other hand, the cytoplasmic signaling pathways for both TLR and Toll are highly conserved. Activation of both pathways leads to an assembly of adaptor proteins recruited to the Toll receptor, which propagates the signal to phosphorylate the IκB complex and release the NF-κB transcription factor to activate the induction of critical AMPs in insects and pro-inflammatory cytokines in mammals (Engstrom et al., 1993; Petersen et al., 1995; Meng et al., 1999). In both systems, the desired outcome is the induction of peptides that can amplify the immune response in order to promote clearance of the pathogen either directly through the innate system in insects or indirectly by activating the adaptive system in mammals.

In the mammalian system, ten TLRs have been identified and studied for specific binding tendencies (Medzhitov et al., 1997; Rock et al., 1998). While certain TLRs function to bind very specific ligands, others bind to a variety of substrates and can converge their binding into many intracellular signals. The TLR most highly associated with Gram-negative LPS sensing is TLR4. It was identified as the receptor responsible for LPS sensing in mammals when a point mutation within the cytoplasmic TIR domain of the protein abolished signaling through the TLR pathway for specific mouse strains (Poltorak et al., 1998). Constitutive activation of TLR4 induces constant activation of NF-κB, which further confirms the link between the receptor and the homologous Toll pathway (Kirschning et al., 1998). TLR4 functions by contacting the pathogens directly in obligate conjunction with the aid of two associated proteins, MD2 (Lymphocyte Antigen 96) and Cluster of Differentiation 14 (CD14; Pugin et al., 1994; Shimazu et al., 1999). This result in the recruitment of MyD88, which in turn recruits the kinases IRAK1 and IRAK4 together with interferon regulatory factor 5 (IRF5), receptor-interacting protein 1 (RIP1), and TNF receptor associated factor 6 (TRAF6). TRAF6 functions to produce a poly-ubiquitin scaffold on itself, which recruits a complex of transforming growth factor beta (TGF-β) activated kinase 1 (TAK1) and TAK1-associated binding proteins 1 and 2 (TAB1, TAB2). TAK1 activation phosphorylates and activates the IKK complex that phosphorylates the IκB kinase (IKK) that leads to its degradation and the liberation of NF-κB to move to the nucleus and activate transcription of a large number of genes that regulate the inflammatory and immune responses (**Figure 1B**).

Transfection of TLR4 into experimental cell lines failed to induce an antimicrobial response to the presence of LPS, which led to a search for additional receptor molecules that act together with TLR4 (Kirschning et al., 1998). RP109 is an extracellular receptor homologous to the *Drosophila* Toll receptor that associates with surface molecule, MD-1, which participates in protein-protein interactions (Miyake et al., 1998). This previous knowledge led to the detection of a similar surface protein, MD2, which associates physically with TLR4 on the cell surface and increases NF-κB activation by threefold in contact with TLR4 (Shimazu et al., 1999). MD2-knockdown mutant mice are very susceptible to Gram-negative bacteria infections due to an inability to recognize LPS and a blockage of TLR4 translocation to the cell surface of leukocytes (Nagai et al., 2002). These studies supplied ample evidence to show that MD2 is a protein that is necessary for the LPS-recognition complex in mammals, as well as an obligate component for the expression of TLR4 on the cell surface of leukocytes, such as macrophages and neutrophils.

Before being associated with the TLR4 LPS-sensing complex, CD14 was identified as a leukocyte PRR that induces the production of inflammatory cytokines in response to LPS (Wright et al., 1990; Pugin et al., 1994). Analysis of a recessive mutation, *Heedless*, which is characterized by a premature stop codon within the gene encoding CD14, indicated that *CD14* mutants would induce a significantly reduced TLR4 signal through MyD88, a TLR adaptor protein, in response to LPS (Jiang et al., 2005). This evidence points to the necessity of a TLR4/CD14/MD2 complex that is activated upon detection of Gram-positive bacteria, which is now known to be responsible for rapid TNFα cytokine induction during infection (Borzecka et al., 2013). While a direct homolog for MD2 has not been found in insects, CD14 may be the mammalian homolog to GNBP1 in insects due to their similarity in membrane anchorage through GPI (Kim et al., 2000). The result of these interactions between TLR4, CD14, and MD2 leads to the synthesis of pro-inflammatory cytokines, which are released from leukocytes in response to bacterial infection in order to recruit more phagocytic cells and lymphocytes to aid in controlling the pathogen and ultimately inducing the formation of immunological memory.

In comparing these two systems, Toll and TLR4, the construction of protein complexes in association with the signaling TIR domain are equally critical for both pathways and are required for AMP or cytokine production, which is the hallmark of each innate signaling immune response. The major difference is that TLR4 serves as a direct binding site for pathogen contact, while Toll requires circulating PGRPs to bind the pathogen and induce serine protease activation. While TLR4 and Toll share a high degree of structural homology, their functions have adapted to fit the needs of the organism. Toll integrates the signal from three extracellular proteins to mount an exacerbated response to a pathogen, while TLR4 rapidly binds PAMPs directly and releases cytokines for further innate amplification and adaptive immune activation.

## TOLL AND TLR PATHWAY RECOGNITION RECEPTORS FOR FUNGAL DETECTION

In addition to recognizing Gram-positive bacteria, the Toll pathway in insects also responds to infections by entomopathogenic fungi. Fungal infection of wild-type *Drosophila* induces rapid expression of drosomycin, an AMP with antifungal properties, but mutants for *Spaetzle*, *Toll*, *Tube*, and *Pelle* show reduced levels of drosomycin upon fungal infection (Fehlbaum et al., 1994; Lemaitre et al., 1996). Later studies have demonstrated that in fungal-infected *Toll* mutants, not only is drosomycin not expressed, but flies are also highly susceptible to the fungi with most flies dying within the first 24 h (Lemaitre et al., 1997). Together, these results indicate the existence of an antifungal pathway that relies on intracellular Toll signaling and the production of the AMP drosomycin.

Although Gram-positive bacteria are sensed through a complex of PRGP-SA, PGRP-SD, and GNBP1, these proteins do not function in the recognition of fungi. The potential of a separate receptor system for fungi was realized when *Drosophila* mutants for *seml*, the gene encoding PGRP-SA, did not impede Toll activation during fungal infections (Michel et al., 2001). In addition to GNBP1, another member of the GNBP family, GNBP3 in *Drosophila* was characterized for its homologous structure to lepidopteran β-(1,3)-glucan recognition proteins (Kim et al., 2000). Just as PG components are ligands for PGRP recognition, β-glucan structures are known fungal cell wall components that act as signals that activate the antifungal functions of Toll. Analysis of GNBP3 mutants has shown that these flies have significantly reduced expression of drosomycin. Recombinant GNBP3 has strong binding affinities to polysaccharide and β-(1,3)-glucan components of the fungal cell wall, even in null *Toll* mutants, indicating that this receptor functions in the hemolymph prior to Spaetzle cleavage and Toll activation (Gottar et al., 2006). Fly mutants for *GNBP3* are equally susceptible to Gram-positive bacteria as wild-type flies, indicating that GNBP3 does not act in cooperation with the PGRP-SA and GNBP1 proteins. This evidence has established GNBP3 as the major receptor for fungal infections in insects.

Curiously, *GNBP3* mutants block drosomycin production only when dead fungal spores are injected to the flies, whereas the AMP expression in response to live spores is unaltered. This led to the discovery of the serine protease Persephone (psh), which is activated upon recognition of virulence factors produced by live fungi (Gottar et al., 2006). *Psh* mutants are highly susceptible to fungal infection, which is accompanied by low levels of drosomycin production. Interestingly, inactivation of three alternate serine proteases, Gastrulation, Easter, and Snake, does not affect drosomycin expression due to the ability of psh to cleave Spaetzle and activate Toll signaling. Under normal conditions, the serine protease inhibitor, Necrotic, inhibits psh. This relationship was established when necrotic mutants showed constitutive psh-dependent activation of Toll that led to spontaneous melanization, cellular necrosis, and shortened fly lifespan (Ligoxygakis et al., 2002).

Persephone and GNBP3 act exclusively to detect antifungal infection and link their activation into proteolytic serine protease cascades that lead to the cleavage of Spaetzle, which induces Toll-mediated AMP transcription through the nuclear translocation of *Drosophila* DIF, an NF-κB homolog. In mammalian fungal detection systems, the TLRs function as pathogen recognition molecules by interacting with the pathogens directly. TLR2 in mammals is required for induction of inflammatory reactions in response to fungal infection based on the rapid recruitment of fungi and fungal fragments to the macrophage phagosome (Underhill et al., 1999). Upon recruitment, TLR2 dimerizes complex with either TLR6 or TLR1 to bind directly to zymosan ligands that are present on fungal surfaces, leading to the activation of NF-κB signaling that results in the production of pro-inflammatory cytokines, mainly TNFα (Ozinsky et al., 2000). Further studies used *MyD88* and NF-κB mutants to demonstrate the homology between TLR2 and the Toll signaling pathway. Without the intracellular components MyD88 and NF-κB, TLR2 activation by zymosan ligand binding does not lead to the production of TNFα (Young et al., 2001). Without cytokine secretion, the mammalian immune system is unable to induce the inflammatory processes that lead to the ultimate production of antibodies during fungal infections.

The primary recognition receptors represent the molecules of greatest divergence between the insect and mammalian immune pathways. While insects use PGRP-SA, PGRP-SD, GNBP1, GNBP3, and Psh, mammals use TLR2, TLR4, CD14, and MD2. TLR2 and TLR4 are highly homologous to insect Toll with respect to their characterization as Type I transmembrane proteins consisting of leucine-rich repeat domains, and a cytoplasmic TIR domain that promotes recruitment of adaptor proteins (Medzhitov et al., 1997). But while these structures are homologous, the physiological functions within insects and mammals are very different; direct vs. indirect interactions with PAMPs. TLR2 does associate with other innate signaling molecules for responses to phagocytosed microbes, but with regard to fungal infection, dimerization of TLR2 with TLR1 or TLR6 is the only requirement. The extracellular steps from pathogen recognition to activation of Toll in insects, such as the serine protease cleavage of Spaetzle, do not function in mammals and are not required for activating leukocytes, highlighting a key evolutionary divergence.

## IMMUNE DEFICIENCY AND TUMOR NECROSIS FACTOR SIGNALING PATHWAY RECOGNITION RECEPTORS

**FIGURE 2:**
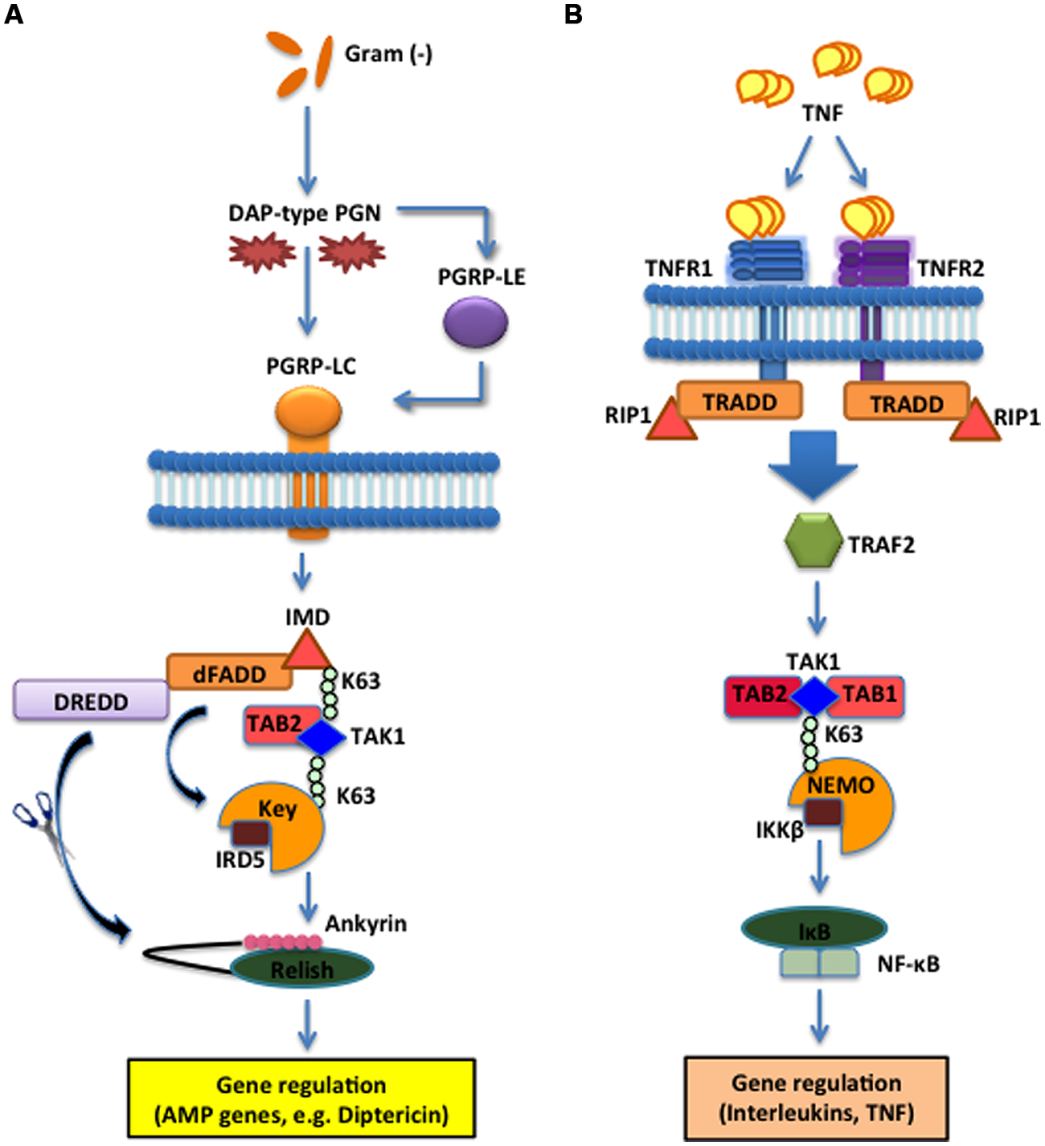
**The Imd pathway in the fruit fly and the TNF pathway in the mouse. (A)** The *D. melanogaster* Imd signaling pathway is activated upon direct binding between PGRP-LC and meso-diaminopimelic acid (DAP)-type PG of Gram-negative bacteria and certain Gram-positive bacilli. The intracellular adaptor protein Immune deficiency (Imd) interacts with the *Drosophila* Fas-associated death domain (dFADD) and the Death related ced-3/Nedd2-like caspase (DREDD) that cleaves Imd, which is then activated by K63-ubiquitination. This leads to the activation of the TAK1 and TAB2 complex that in turn activates the IKK signalosome, which is composed of Immune Response Deficient 5 (IRD5) and Kenny (Key). Relish is subsequently cleaved by DREDD. As a result, the Rel DNA-binding domain is released from the C-terminal ankyrin-repeat/IκB-like domain, and translocates to the nucleus to induce transcription of antimicrobial peptide (AMP) genes, such as Diptericin. **(B)** In *Mus musculus*, TNF trimers bind and activate the transmembrane receptors R1 and R2 (TNFR1 and TNFR2) that recruit Tumor necrosis factor receptor type 1-associated DEATH domain protein (TRADD), receptor-interacting protein 1 (RIP1) and TNF receptor-associated factor 2 (TRAF2). The latter employs the Transforming Growth Factor beta (TGF-β) activated kinase 1 (TAK1) (whose activity is directly regulated by K63-linked polyubiquitination) and TAB1 and TAB2 complex to phosphorylate and activate the IKK signalosome, which phosphorylates IκB that dissociates from NF-κB. NF-κB translocates to the nucleus to induce expression of several genes that participate in inflammation and immunity.

The Imd effect was identified by the severely impacted immune phenotypes produced by *Drosophila* mutants in the intracellular adaptor protein Imd, which interacts with the *Drosophila* Fas-associated death domain ortholog (dFADD) that binds to Death related ced-3/Nedd2-like caspase (DREDD). Imd is cleaved by DREDD and is subsequently activated by K63-ubiquitination (Paquette et al., 2010). Fly mutants for Imd are characterized by the lack of AMP production and an increased susceptibility to Gram-negative bacteria (Lemaitre et al., 1995). Similar to the mechanistic structure of the Toll pathway, Imd integrates the activation signal through intracellular adaptor proteins that converge on inducing the nuclear translocation of Relish, another homolog of NF-κB. Relish is activated by Immune Response Deficient 5 (IRD5; IKKβ homolog) and Kenny (Key; IKKγ homolog) which form the fly IKK signalosome that is phosphorylated and activated by the TAK1/TAB2 complex (Kleino and Silverman, 2014). The caspase DREDD cleaves Relish, removing the C-terminal inhibitor ankyrin-repeat/IκB-like domain, which remains in the cytoplasm, which allows the Rel DNA-binding domain (Rel68) to translocate to the nucleus where it induces the transcription of target genes. Target genes in the Imd pathway code for AMPs such as Diptericin and Cecropin, which act against Gram-negative bacteria (**Figure 2A**). The insect Imd pathway is homologous to the mammalian TNF signaling pathway based on the intracellular mechanisms, although the recognition receptors are different (Lemaitre and Hoffmann, 2007). In mammals, trimeric forms of TNF bind and activate TNF receptor 1 and 2 (TNFR1, TNFR2) cytoplasmic receptors that recruit a cytoplasmic complex composed of tumor necrosis factor receptor type 1-associated DEATH domain protein (TRADD), RIP1 and TRAF2 that activates the IKK signalosome via the TAK1/TAB1/TAB2 complex leading to NF-κB translocation and transcriptional induction of genes that modulate inflammation and immune function (**Figure 2B**). Imd is highly homologous to the mammalian RIP1, which binds to the cytoplasmic domain of the TNFR (Hsu et al., 1996). Both Imd and TNFR signaling induce the recruitment of adaptor proteins through death domain interactions, which results in a phosphorylation cascade to induce NF-κB-mediated transcriptional activation of antibacterial molecules and cytokines (Georgel et al., 2001). Therefore, while the receptor mechanisms are not inherently homologous, they are critically important to the integration of information that is transduced to their homologous intracellular counterparts, which illuminates key parallels between insect and mammalian innate immune systems.

Based on the intracellular nature of Imd, it became apparent that an extracellular recognition receptor would be imperative for relaying information from the hemolymph to the intracellular Imd pathway in response to Gram-negative bacteria. Once PGPR-SA was identified as a PRR for the Toll pathway, researchers began to analyze the other 13 PGRPs in insects for their involvement in activating the Imd pathway. A mutant strain for PGRP-LC shows a loss-of-function immune phenotype that is similar to mutants for the Imd pathway, and is characterized by reduced survival in response to Gram-negative bacteria while the survival in response to Gram-positive bacteria is unaffected (Gottar et al., 2002). Similar to the specificity of Toll pathway receptors to Lys-type PG, PRGP-LC is able to identify and bind to DAP acid, found in the stem peptide, and 1,6-anhydro forms of MurNAc found in the glycan chain, which are not present in Gram-positive bacteria. These structures are exclusive to Gram-negative bacteria and have been identified as the minimal requirements for PGRP-LC binding (Stenbak et al., 2004). PGRP-LC communicates exclusively with the Imd pathway, because over expression of PGRP-LC also leads to constitutive expression of the Imd-specific AMP, diptericin. Expression of diptericin is only recorded when Imd is also present, which leads to the conclusion that PGRP-LC functions upstream of the intracellular cascade. PGRP-LC is characterized as a long PGRP that contains a single transmembrane domain, an N-terminal cytoplasmic domain and extracellular PGRP domains that are used to integrate and transmit signals (Werner et al., 2000). In addition, these cytoplasmic domains are found in three alternative splice isoforms (LCa, LCx, and LCy) that heterodimerize with each other to recruit Imd through death domain interactions that propagate the cytoplasmic signal (Choe et al., 2002; Lim et al., 2006). These characteristics make PGRP-LC the key transmembrane receptor for Imd activation and Gram-negative bacteria detection.

While PGRP-LC is necessary for Imd pathway activation, mutant phenotypes are not as severely susceptible to bacterial infection as loss-of-function mutants for *Key*, which encodes the insect homolog of IKKβ/NEMO and is part of the intracellular Imd pathway (Silverman et al., 2000; Lu et al., 2001; Ertürk-Hasdemir et al., 2009). *Key* mutants are highly susceptible to infection because the IKK complex provides the phosphorylation signal to the IκB subunits to release the NF-κB Rel domain, which drives transcriptional activation. Once the activation is transduced into the cytoplasm, the signaling pathway is very linear, which allows the susceptibility of *Key* mutants to be evaluated based on the inability of the Imd pathway to produce AMPs. Therefore, because the *PGRP-LC* mutants show a decreased impact, this implies that additional receptors converge on the linear cytoplasmic pathway and account for the reduced susceptibility. This conclusion led to the identification of PGRP-LE that is exclusively involved in the detection of DAP-type bacteria for the Imd pathway.

Additional studies identified PGRP-LE through a gain-of-function screen indicating that this PGRP is able to induce AMP synthesis without bacterial infection (Takehana et al., 2004). Null mutants for PGRP-LE have wild-type resistance to Gram-positive bacterial infections by *B. subtilis* and *Micrococcus luteus*, but are highly susceptible to Gram-negative infections of *E. carotovora*. Double mutants for *PGRP-LC* and *PGRP-LE* show an even greater reduction in resistance to Gram-negative bacteria than Imd mutants, indicating that these proteins act synergistically in response to DAP-type bacteria. To confirm a link to the Imd pathway, this double mutant has been compared to a double mutant for *Relish*, the Imd homolog NF-κB transcriptional activator, to show that both mutants are equally susceptible to Gram-negative bacterial infection. This synergism works both within the hemolymph and cytoplasm due to intracellular and extracellular forms of PGRP-LE. In the hemolymph, PGRP-LE functions as a circulating receptor, similar to PGRP-SA, by binding to extracellular bacteria, presenting the indicator of infection to the transmembrane receptor, PGRP-LC, and inducing downstream signaling leading to Imd (Kaneko et al., 2006). PGPR-LE is also found in the cytoplasm, where it binds to intracellular Gram-negative bacteria and interacts with the cytoplasmic N-terminal region of PGRP-LC, which also activates the death domain of Imd (Kaneko et al., 2006). The structure of PGRP-LE displays a similar PG -binding groove seen in PGRP-SA including the critical residue, Ser158. In PGRP-LE binding grooves, Arg254 provides an ionic neutralization interaction between the receptor and the carboxyl group of DAP-type PG (Lim et al., 2006). Mutations affecting this arginine show severe impairment of PGRP-LE binding to Gram-negative bacteria, indicating that this position is a critical point of interaction. Not only has this evidence confirmed PGRP-LE as the additional recognition protein receptor of the Imd pathway, but it has also made PGRP-LE the only known intracellular recognition molecule found in *Drosophila*.

Comparing the insect and mammalian Toll pathways shows that PGRPs are necessary for the insect response, but are not involved in the mammalian response. This characteristic difference is maintained in the homologous Imd and TNF pathways as well. As described above, insects rely on PGRP-LC and PGRP-LE for these actions in targeting and binding to DAP-type bacteria and eliciting an antimicrobial response. The TNF pathway acts in mammalian response to bacterial and viral infections functions without the involvement of PGRPs. Instead, TNFR1 is the primary transmembrane receptor for soluble TNFα, which is a mammalian cytokine released during bacterial and viral infections. Upon binding to the cytokine, TNFR1 transmits a signal through its membrane domain to RIP1, which interacts with adaptor proteins homologous to *Drosophila* counterparts, to induce nuclear translocation of NF-κB and cytokine synthesis. Mouse *TNFR1* mutants alone do not produce immune deficiencies, unlike those observed in severely compromised *PGRP-LC* mutants. When TNFR1 is mutated together with the mammalian NF-κB gene *RelA*, TNFR1/RelA-deficient mutant mice exhibit severe susceptibility to bacterial infection, hematopoietic defects and significant reductions in neutrophil recruitment to sites of injury and injected bacteria (Alcamo et al., 2001). This evidence indicates that TNFR1 interaction with NF-κB is essential for TNFR1 pathway activity to maintain transcriptional activation of inflammatory cytokines that induce leukocyte recruitment. Like Imd, these pathways integrate signals from pathogens or molecules released upon recognition that lead to the activation of a transmembrane receptor, either PGRP-LC in conjunction with PGRP-LE in insects, or TNFR1 in mammals. These proteins respond to different signals, such as DAP-type PGs from bacteria or cytokines released from leukocytes, but the cellular integration of the information is homologous. For insects, the Imd pathway is activated mainly in response to Gram-negative bacteria by selectively binding to PG structures through ionic interactions and triggering the Relish-mediated transcription of antibacterial peptides. TNFR1 incorporates signals from active macrophages, which release cytokines to induce TNFR1-mediated signal transduction to produce more cytokines via NF-κB transcriptional activation. Similar to the intrinsic differences between insect and mammalian Toll pathways, the Imd pathway is critical for targeting and eliminating bacteria in insects, and the TNFR1 pathway is critical for inducing intermediate inflammatory immune responses while the adaptive system can prepare antibodies for further pathogen elimination.

## CYTOKINE RECEPTORS IN JAK/STAT SIGNALING

The JAK/STAT pathway was originally discovered in mammals and subsequently in insects (Brown et al., 2001). It is responsible for the innate immune response to septic injury and infection against bacterial and viral pathogens (Morin-Poulard et al., 2013; Myllymäki and Rämet, 2014). The mammalian JAK/STAT pathway was first identified during screens of interferon-induced activity, in which JAK was upregulated and consequently tied to the intracellular signaling of interferon and cytokine receptors. Interferon-mediated activation of further signaling is dependent on the activity of the tyrosine kinase, JAK, which induces phosphorylation of STAT in order to activate transcription of genes that are critical to the inflammatory response (Lee et al., 1997). In comparison to insect Toll and Imd pathways, in which the intracellular components were characterized before those in mammals, the cytokine receptors associated with mammalian JAK/STAT were isolated and later found to be the recognition receptors needed for activation of this pathway (Lutticken et al., 1994; Stahl et al., 1994). This led to the identification of homologous pathways in *Drosophila*, involving JAK and STAT homologs, Hopscotch, and Marelle, respectively (Hou et al., 1996). The *Drosophila* JAK/STAT pathway functions in both immune and developmental processes. In *Hop* and *Marelle* mutants, *Drosophila* exhibits larval lethality, improper abdominal segmentation, and wing-vein abnormalities (Yan et al., 1996). Mutations in JAK/STAT pathway components of insects affect the function of hematopoiesis and the production of hemocytes, whereas mutations in JAK/STAT pathway components of mammals interfere with the activation of cellular immunity that is regulated through myeloid progenitor cells (Luo et al., 1997; Agaisse et al., 2003).

**FIGURE 3:**
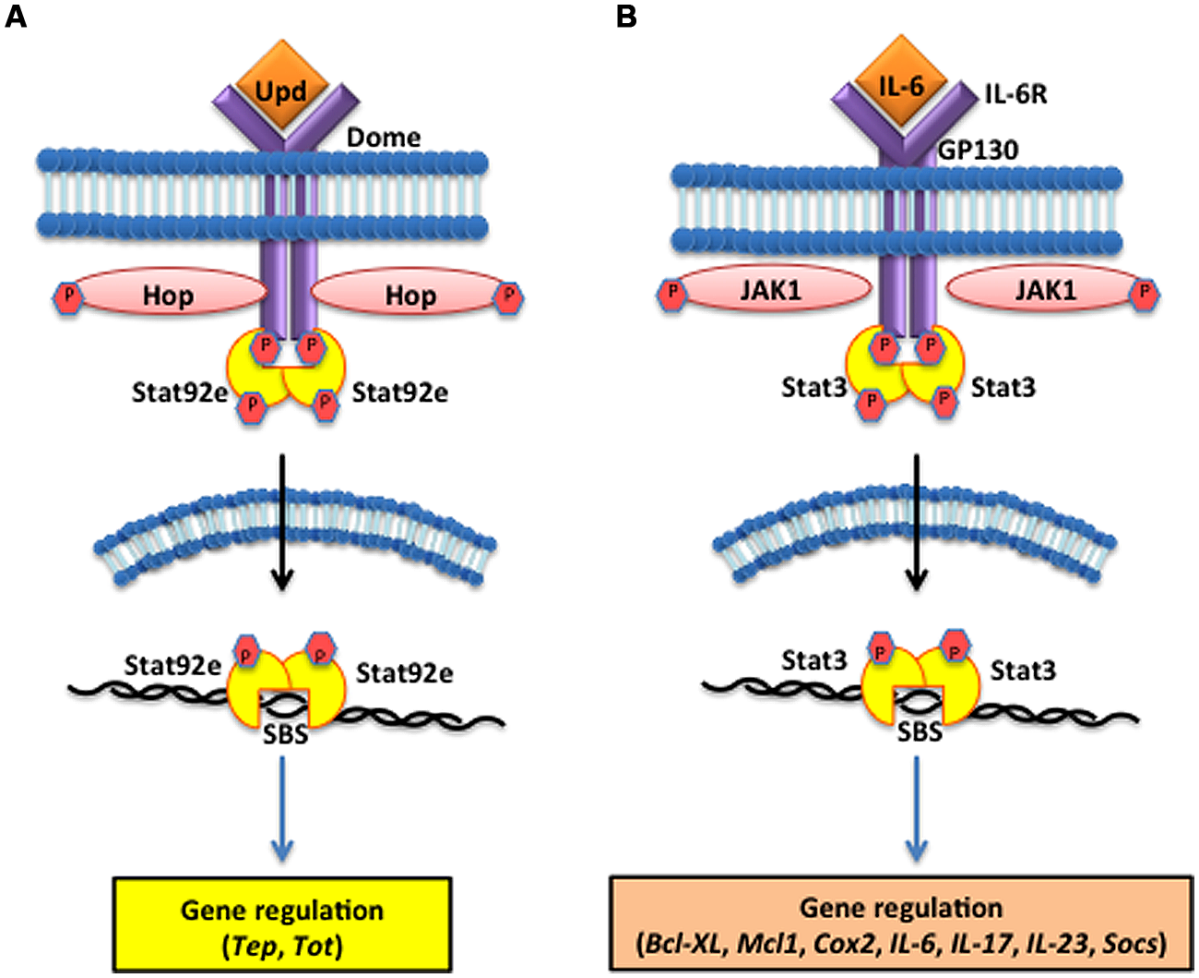
**The Janus kinase/signal transducers and activators of transcription (JAK/STAT) pathway in the fruit fly and the mouse. (A)** The *D. melanogaster* JAK/STAT cytokine receptor, Domeless (Dome) is activated upon binding the Unpaired (Upd) cytokines, which causes the JAK tyrosine kinase Hopscotch (Hop) to phosphorylate itself and the cytoplasmic tail of Dome. The signal-transducer and activator of transcription at 92E (Stat92e) bind to the phosphotyrosines on the receptor, and are phosphorylated by Hop. Stat92e dissociate from the receptor, dimerize, move to the nucleus, and induce the transcription of Thioester-containing protein genes (Teps) and Turandot (Tot) stress genes. **(B)** In *Mus musculus*, interleukin-6 (IL-6) binds to its receptor (IL-6R) and activates the Glycoprotein GP130 via the JAK1/JAK2 kinases. STAT3 activation is dependent upon tyrosine phosphorylation, which induces dimerization via reciprocal phosphotyrosine-SH2 (Src homology domain 2) interaction between two STAT3 molecules. Activated STAT3 transcription factors translocate into the nucleus where they bind to consensus promoter sequences and cause the transcriptional induction of target genes, such as B-cell lymphoma-extra large (Bcl-xl), myeloid cell leukemia 1 (Mcl-1), cytochrome c oxidase II (Cox2), ILs [IL-6, IL-17, IL-23, Suppressor of cytokine signaling (Socs)].

The *Drosophila* JAK/STAT cytokine receptor, Domeless (Dome), was identified through bipartite complementation assays showing that JAK homodimerization is abolished in the absence of Dome (Brown et al., 2003). Structural analysis characterized Dome as a transmembrane protein containing extracellular fibronectin type III domains with high homology to mammalian type I cytokine receptors of the interleukin-6 (IL-6) family, such as gp130 and IL-6R (Brown et al., 2001). The Unpaired (Upd) ligands are secreted upon injury or infection (**Figure 3A**). These glycosylated molecules were originally implicated in mutants that displayed small eyes and abnormal wing development phenotypes, but were later shown to induce tyrosine phosphorylation of Hop, the *Drosophila* homolog of JAK (Harrison et al., 1998). Loss-of-function *Upd*, *Jak*, and *Stat* mutants show similar phenotypes, leading to the conclusion that Upd molecules serve as the ligand for receptors that induce JAK/STAT signaling. Phosphorylation of the *Stat92e* transcription factors results in their dimerization and translocation into the nucleus where they bind to target sequences to induce effector gene expression. These include *Turandot A* (*TotA*) and complement-like thioester proteins, in insect hemocytes, that are dependent on Dome/upd3 signaling for expression (Lagueux et al., 2000; Agaisse et al., 2003).

Activation of the mammalian JAK/STAT pathway is triggered by ILs, interferons, and growth factors released by phagocytic leukocytes responding to infection or injury to the host (Parganas et al., 1998). Mammals have a large variety of cytokine receptors but JAK/STAT complexes only associate with type I and type II cytokine receptors, which bind to a variety of ILs and interferons (Bazan, 1990; Marchetti et al., 2006). These receptors span the cell membrane of leukocytes and lymphocytes and extracellular domains share common amino acid motifs in type I cytokine receptors, but are more diverse in type II. Interferon binding promotes antiviral responses while binding IL-6 induces immune response and stress related genes. IL-6 binding to IL-6R activates the signal transducer glycoprotein 130 (GP130) through the JAK1/JAK2 threonine kinases that cause the nuclear translocation of the STAT3 transcription factor and subsequent DNA binding and gene regulation (**Figure 3B**); (Darnell et al., 1994; Rawlings et al., 2004; Li, 2008; Ghoreschi et al., 2009). However, insect JAK/STAT only associates with a single cytokine-like receptor and integrates signaling based on the binding of three ligands, known as Upd molecules (Upd1, Upd2, and Upd3; Harrison et al., 1998; Hombría and Brown, 2002; Hultmark and Ekengren, 2003). In conclusion, the JAK/STAT pathway of insects is linear and involves one receptor, three ligands, and many transcriptional outputs. Mammalian JAK/STAT pathways involve a multitude of type I and type II cytokine receptors, numerous cytokines, and synthesis of diverse peptide synthesis. This discrepancy in the number of receptors reflects how mammals amplify the inflammatory response to a greater degree than insects in order to induce adaptive immunity. But in terms of Dome comparisons to the Type I cytokine receptors found in the mammalian JAK/STAT pathway, these receptors recognize ligands, bind, and transduce signals in a homologous manner.

## CONCLUDING REMARKS

The insect innate immune system is capable of mounting a robust response upon infection with bacteria and fungi through the release of AMPs, cytokines, and many other immune-response proteins. The insect immune signaling pathways described in this review rely on the presence of properly functioning extracellular and intracellular PGRPs, GNBPs, cytokine receptors, and their associated protein complexes that initiate signaling. In particular, the insect immune response against Gram-positive bacteria is orchestrated through the individual and associative functions of PGRP-SA, GNBP1, and PGRP-SD, which are able to recognize Lys-type bacteria, bind directly to the fragmented PG monomers and polymers and induce a serine protease cascade in the hemolymph that induces Spaetzle/Toll binding to trigger intracellular signal propagation. GNBP3 and Psh also induce and facilitate the serine protease targeting Spaetzle, Toll activation, and subsequent NF-κB-mediated AMP synthesis by binding specifically to fungal β-glucan structures. On the other hand, mammalian TLRs function directly as the PAMP recognition receptors that bind and transduce the information on pathogen detection to the intracellular signaling complex. More specifically, mammalian TLR4 and TLR2 are the direct homologs of insect Toll, which bind to LPS and zymosan components, respectively. TLR4 also shows similar abilities to PGRP-SA for forming complexes, in that the protein requires CD14 and MD2 to aid in LPS sensing mechanisms, however, the structures of PGRPs and TLRs are very different and lead to significantly different physical interactions with the PAMPs.

There are also similarities between the insect Imd pathway and the mammalian TNF pathway. Insects rely on PGRP-LC and PGRP-LE to target specifically DAP-type PG structures that are signature PAMPs of Gram-negative bacteria and relay the information through Imd-specific adaptor proteins to the transcription factor Relish for inducing AMP synthesis. The mammalian TNF pathway is activated by the TNFR1 protein, which is very different structurally from the PGRP receptors of insects. TNFR1 recognizes cytokines, mainly TNF-α, which is released from leukocytes in order to recruit inflammatory cells to the site of injury and infection. However, the homology between the TNF pathway and the Imd pathway is based on the intracellular branching of the signaling pathway leading to the transcriptional activation of genes that are mediated by NF-κB and JNK homologs, which demonstrates clear conservation between the insect and mammalian systems. The receptors, TNFR1, and the PGRPs are the most structurally and functionally divergent of the three pathways described in this review. One of the major differences between the insect and mammalian pathways is that the mammalian pathways are activated by cytokines that stimulate an amplified inflammatory response and can lead to an adaptive response, whereas the insect pathways detect the presence of the pathogen, which activates the production of antibacterial or antifungal molecules that simply act to clear the Gram-negative bacteria as quickly and efficiently as possible. Finally, the JAK/STAT pathways of insects and mammals show the greatest homology of the three signaling pathways discussed herein. Insect and mammalian JAK/STAT pathways are structurally and functionally homologous to bind to interferon and IL molecules and stimulate the downstream phosphorylation of JAK, dimerization of STAT, and activation of pro-inflammatory molecules that can act to inhibit microbial infections, repair septic damage, or induce the production of antibodies.

Overall, signal transduction is a powerful mechanism that is employed by both the insect and mammalian innate immune systems. While the homologous intracellular mechanisms are essential for these functions and have remained unchanged between these two groups of animals, the receptors that function in recognizing and targeting the infectious molecules are of equal importance and show both conserved and quite different attributes between mammals and insects. Through the evaluation and comparison of these receptors, a greater understanding is achieved for the common immunological goals of each organism, which encompasses both the conservation of innate proteins, and the divergences. The evolutionary variations in the mammalian pathways are all connected to the activation and functions of the vertebrate adaptive immune system. For mammals, eradicating the pathogen is essential, but in certain pathways, such as TNF and JAK/STAT, slight divergences from the insect systems show new obligations that serve to amplify cellular signals and stimulate the adaptive system that have become the staple of mammalian immunity. Although insects lack an adaptive immune system that is homologous to the V(D)J recombination mechanisms, PRRs are entirely responsible for the integration of relevant information for appropriate immunological responses to classes of pathogens with the outcome of efficient reactions of insects to microbes that has proven to be an exceptional strategy for the their survival and evolutionary success.

## Conflict of Interest Statement

The authors declare that the research was conducted in the absence of any commercial or financial relationships that could be construed as a potential conflict of interest.
